# ♣Evaluation of clinicopathological profiles and development of a risk model in renal epithelioid angiomyolipoma patients: a large-scale retrospective cohort study

**DOI:** 10.1186/s12894-022-01101-9

**Published:** 2022-09-12

**Authors:** Aihetaimujiang Anwaier, Wen-Hao Xu, Xi Tian, Tao Ding, Jia-Qi Su, Yue Wang, Yuan-Yuan Qu, Hai-Liang Zhang, Ding-Wei Ye

**Affiliations:** 1 Department of Urology, Fudan University Shanghai Cancer Center, No. 270 Dong’an Road, Shanghai, 200032 People’s Republic of China; 2 Department of Oncology, Shanghai Medical College, Fudan University, Shanghai, 20032 People’s Republic of China; 3 Department of Urology, Southern Medical University Affiliated Fengxian Hospital, Shanghai, 201499 People’s Republic of China

**Keywords:** Renal epithelioid angiomyolipoma, SMA, Ki-67, Biomarkers, Predictive model

## Abstract

**Background:**

To identify the malignant potential and prognostic indicators of renal epithelioid angiomyolipoma (eAML), clinicopathological and molecular features as well as the drug efficacy of 67 eAML cases were analyzed.

**Materials and methods:**

Sixty-seven renal eAML patients were enrolled and the immunohistochemical features of these patients were examined. FFPE slides of all patients were re-examined. 21 patients with metastasis received Everolimus 10 mg orally once daily. Responses were evaluated with RECIST criteria by three authors. A risk stratification model was constructed using the following factors: pT3 and pT4, presence of necrosis, mitotic count ≥ 2; the presence of atypical mitoses; severe nuclear atypia, SMA negative, Ki-67 ≥ 10%.

**Results:**

The average percentage of the epithelioid component was 85.6% (range 80–95%). Immunohistochemically, Ki-67 ≥ 10% and negative SMA staining were significantly correlated with malignant characteristics (Ki-67: *p* < 0.001; SMA: *p* = 0.001). Survival analysis suggested that pT3-pT4 stage, presence of necrosis, severe nuclear atypia, presence of atypical mitoses, mitotic count ≥ 2, Ki-67 ≥ 10% and negative SMA expression were significantly associated with poorer PFS and OS (*p* < 0.05). The risk model sufficiently discriminated recurrence/metastasis (AUC = 0.897) and cancer-specific mortality (AUC = 0.932) of renal eAML patients in different risk groups. 21 patients had received Everolimus targeted therapy after recurrence/metastasis. The best response for Everolimus treatment was 8/21 (38.1%) partial responses (PR), 9/21 (42.9%) stable disease (SD) and 4/21 (19.0%) progressive disease (PD).

**Conclusion:**

The risk stratification model could well distinguish eAML patients at high risk of recurrence/metastasis. Everolimus targeted treatment showed good efficacy in patients with recurrence/metastasis.

**Supplementary Information:**

The online version contains supplementary material available at 10.1186/s12894-022-01101-9.

## Introduction

Renal angiomyolipoma (AML) is a relatively rare mesenchymal neoplasm. According to the World Health Organization (WHO) tumor classification, there are two types of renal AML: classic AML and epithelioid AML. Renal epithelioid angiomyolipoma (eAML) is a potentially malignant variant of renal AML [1, 2], accounting for less than 1% of all renal neoplasm [3] and approximately 7.7% of renal AML cases [4]. Unlike classic renal AML, which is composed of various proportions of dysmorphic blood vessels, smooth muscle components, and fat cells, renal eAML consists of at least 80% of epithelioid cells as well [5]. Tumor cells can be polygonal with varying degrees of nuclear atypia, round to oval nuclei, atypical mitoses, and transparent to eosinophilic cytoplasm [6]. Immunohistochemically, typical renal eAML is positive for Human Melanoma Black (HMB-45), MART-1/Melan-A, smooth muscle actin (SMA) and muscle-specific actin myoid markers [7]. Although classic renal AML is considered to be a benign mesenchymal tumor, several studies have demonstrated that renal eAML exhibits aggressive clinical characteristics such as local recurrence and distant metastasis [6, 8–11]. Therefore, renal eAML can be an atypical variant of renal AML or a distinct malignant tumor element [6, 12].

Nevertheless, because of the rarity of renal eAML, there is insufficient data to clarify its clinicopathological characteristics and pathological prognostic predictors. Several case reports and small series studies have investigated renal eAML [13–15], but our understanding of the features and prognosis of renal eAML remain limited. Brimo et al. established a predictive model of renal eAML and reported that four atypical features of renal eAML could accurately categorize 78% of renal eAML cases with malignant characteristics in their series [16]. Subsequently, Nese et al. analyzed 41 cases of pure renal eAML and published risk factors for disease progression. They identified five adverse prognostic parameters and stratified patients into three risk categories [17]. However, the clinicopathological features and risk factors that can predict the prognosis of renal eAML patients need further investigation and validation.

To clarify the clinicopathological characteristics and immunohistochemical (IHC) features of renal eAML and investigate the potential prognostic predictors of renal eAML, we analyzed the clinicopathological data and IHC indexes of a large series of renal eAML cases. Although there are some recognized risk factors for predicting the potential malignancy of renal eAML, for more comprehensive analyses, we attempted to establish a prognostic model to distinguish patients at risk of recurrence or metastasis. Our findings may help identify the malignant potential of renal eAML and its prognostic indicators.

## Methods and materials

### Patients

This study was a retrospective cohort study of 67 renal eAML patients, which were diagnosed from June 2013 to December 2019 at the Department of Urology. According to the WHO definition, all eAML cases enrolled in our cohort consisted of at least 80% of epithelioid cells. The number of cases during the study period determined the sample size. Among them, 4 patients were diagnosed as distant metastasis at the time of diagnosis, so they were excluded from the training cohort but still included in the analysis of treatment response. The study was approved by the institutional review board of our insitution. All cases were independently re-reviewed by two experienced genitourinary pathologists toexclude the misdiagnosed cases and maintain consistency of pathological parameters.

### Clinicopathological data

The clinicopathological parameters of all patients were obtained from electronic medical records or by re-assessed the slides. Tumor size was recorded using the largest tumor diameter. The stage was assessed by combining the clinical and pathological TNM staging according to the American Joint Committee on Cancer (AJCC) TNM staging, 8th edition, 2017. Tumor necrosis was defined as microscopic coagulative necrosis. Epithelioid cells were defined as polygonal cells with clear to deeply eosinophilic cytoplasm, vesicular nuclei, and prominent nucleoli. The degree of nuclear atypia was graded as mild, moderate, or severe. Mild nuclear atypia: Conspicuous nucleoli, nuclear membrane irregularities, and slight variation in nuclear size. Moderate nuclear atypia: Cells are intermediate in size and show enlarged nuclei with moderate pleomorphism and prominent nucleoli. Severe nuclear atypia: Cells are large with abundant eosinophilic cytoplasm, extensive nuclear pleomorphism, and prominent nucleoli [16].The percentage of epithelioid cells and the degree of nuclear atypia were estimated visually in relation to the total tumor areas in the available slides. Atypical mitoses and multinucleated giant cells were assessed in 10 high-power fields (HPFs).

### Immunohistochemical evaluation

All the slides were sectioned using the primary tumor tissue. The IHC profiles of 57 renal eAML patients were assessed using a broad panel of targets: HMB-45, Melan-A, SMA, PNL-2, desmin, CD34, AE1/AE3, CK7, vimentin, TFE3-OPT, PAX8, CD10, S-100, and Ki-67. Positive or negative staining of a target on one FFPE slide and all haematoxylin and eosin (H&E)-stained slides were independently assessed by two experienced genitourinary pathologists. The immunohistochemistry (IHC) staining degree score was graded from 0 to 4, based on the coverage percentage of tumor cells (0%, 1–25%, 26–50%, 51–75%, 76–100%). Staining intensity degrees was ranging from 0 to 3, representing samples with no staining, weak, median and strong, respectively. The overall IHC score (from 0 to 12) was calculated according to the multiply of staining degree score and staining intensity, and 0–2 was defined as negative staining and 3–12 as positive staining.

### Survival assessment

The primary endpoint was overall survival (OS), which was assessed from the date of surgical treatment or needle biopsy to the date of death or last follow-up. Progression-free survival (PFS) was the secondary endpoint and was defined as the length of time from the date of surgery or needle biopsy to the date of progression, second-line treatment, or death, whichever occurred first. Survival curves were established using the Kaplan–Meier method and analyzed by the log-rank test with 95% confidence intervals (95% CIs). To identify independent predictors, hazard ratio (HR) estimates and 95% CIs were calculated using univariate and multivariate Cox logistic regression models.

### Treatment

According to patients’ tolerance, 21 patients have treated with Everolimus 10 mg orally once daily with continues until disease progression, the occurrence of unacceptable toxicity, or death. The inclusion criteria for patients treated with Everolimus are as follows: (1) Age ≥ 18 years; (2) Patients had a histological or cytological diagnosis of at least one metastatic site; (3) patients had a radiologically measurable metastatic disease; 4. Receiving Everolimus as first-line treatment. Dose modification or discontinuation was administered according to the patients’ tolerance. Responses were evaluated with Response Evaluation Criteria In Solid Tumors (RECIST) by three authors. Timing of assessments is at the discretion of the treating physician, usually once every 3 months. Follow-up information was obtained during clinical visits or by telephone.

### Statistical analysis

To maximize the statistical analysis and minimize any bias caused by the missing data, multiple imputation with chained equations was performed using R language to assign missing values.

Correlations between the clinicopathological and IHC parameters of the experimental groups were determined by chi-squared test and independent sample t-test. Continuous variables were reported as means ± SD; categorical variables were reported as the number and percentage of the total population. Evaluations were based on point estimates and 95% CIs. All hypothetical tests were two-sided and *p*-values less than 0.05 were considered significant in all tests.

## Results

### Clinicopathological characteristics

**Fig. 1:**
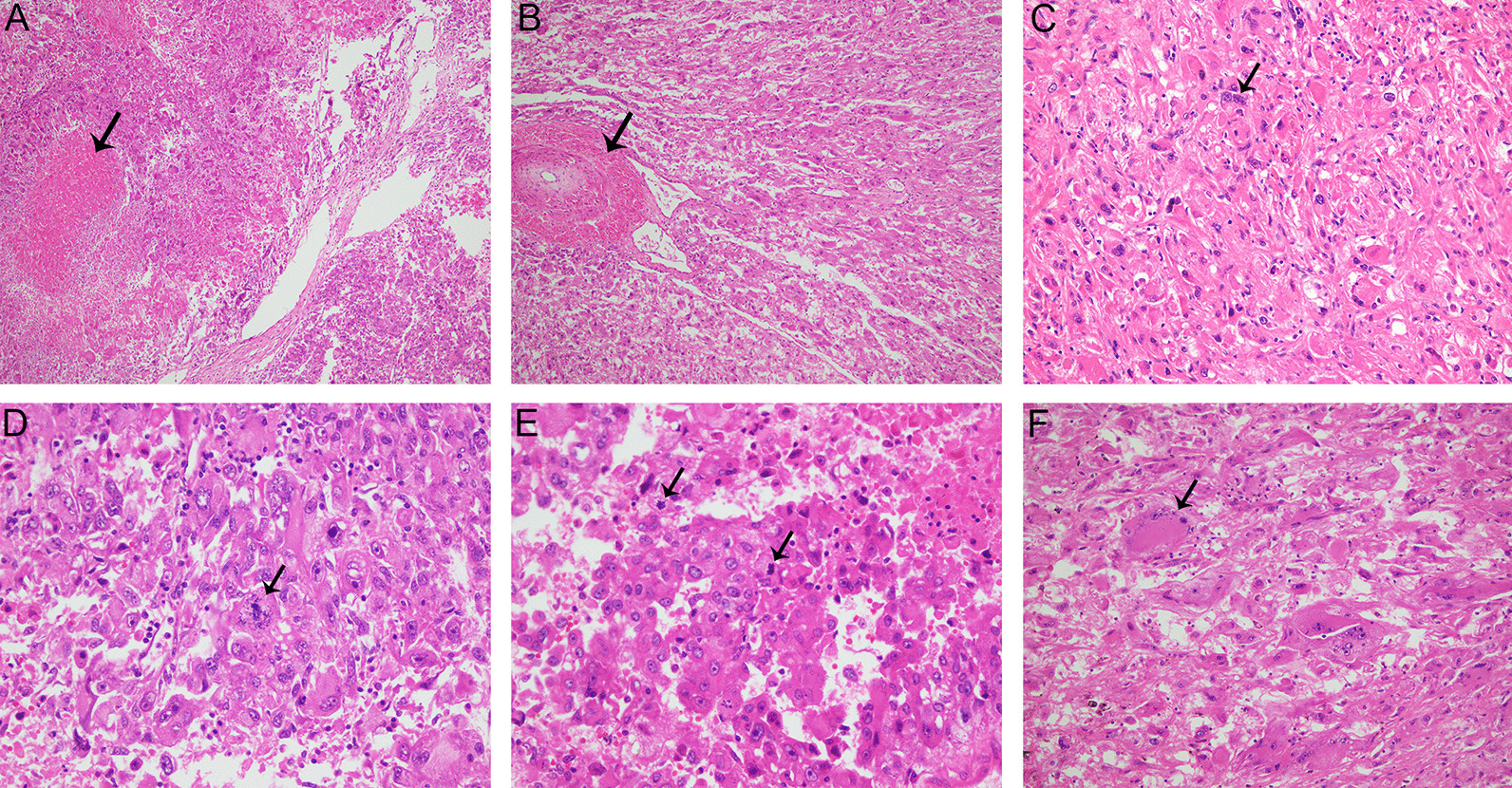
Histopathological features of renal eAML. Haematoxylin and eosin staining. **A** The arrowhead indicates the necrosis in a renal eAML sample. Magnification 100 × . **B** In renal eAML cases, tumors were composed of epithelioid endothelial cells. The arrowhead indicates hyaline degeneration of the vascular wall. Magnification 100 × . As shown by Arrowhead, compared with cells displaying mild nuclear atypia **C**, cells with severe nuclear atypia **(D)** are characterized by their larger size, abundant eosinophilic cytoplasm, and nuclear polymorphism. Magnification 400 × . **E** Renal eAML also displayed atypical mitotic figures (arrows). The higher atypical mitotic figure may associate with malignant behaviors. Magnification 400 × . **F** Arrowhead indicates the Multinucleated giant cell. Magnification 200 ×

Sixty-seven renal eAML patients from our institution were analyzed. The clinicopathological characteristics of patients were shown in Table 1. Over the entire duration of follow-up (median follow-up, 49.5 months, range 8.9–102.5 months), 46 renal eAML patients had no recurrence/metastasis (73.0%) and 17 cases had recurrence/metastasis (27.0%).. The mean age of the study cohort was 40.8 ± 13.7 years and there were 31 men (49.2%) and 32 women (50.8%). The tumor size of recurrence/metastasis cases was significantly larger than that of no recurrence/metastasis patients (6.2 ± 3.5 vs. 11.4 ± 4.9, *p* < 0.001). In addition, patients with recurrence/metastasis were significantly correlated with advanced T (*p* = 0.003) stages. Fifteen cases presented necrosis (Fig. 1A), and the presence of tumor necrosis was significant in the recurrence/metastasis group (7/17, 41.2%) compared with the no recurrence/metastasis group (8/46, 17.4%) (*p* = 0.049). Moreover, four cases presented perinephric fat invasion and one case showed microvascular invasion. Three cases were associated with multiple tumors: a single eAML tumor on the left kidney coexisting with a single eAML tumor on the right lobe of the liver in one case; multiple eAML tumors on the right kidney in one case; and a single eAML tumor on both kidneys in one case.

**Table 1:** Comparison of clinicopathological characteristics of 63 renal eAML patients from the FUSCC cohort

Variable	Entire group (n = 63)	No recurrence/metastasis (n = 46)	Recurrence/metastasis (n = 17)	*p* value
Age (years, mean ± SD)	40.8 ± 13.7	40.5 ± 13.1	42.0 ± 15.7	0.791
Size (cm, mean ± SD)	7.6 ± 4.5	6.2 ± 3.5	11.4 ± 4.9	** < 0.001**
Sex (n, %)				0.607
Male	31 (49.2)	22 (47.8)	9 (52.9)	
Female	32 (50.8)	24 (52.2)	8 (47.1)	
Laterality (n, %)				0.163
Left	28 (44.4)	18 (39.1)	10 (58.8)	
Right	35 (55.5)	28 (60.9)	7 (41.2)	
pT stage (n, %)				**0.003**
T1–T2	44 (69.8)	37 (80.4)	7 (41.2)	
T3–T4	19 (30.2)	9 (19.6)	10 (58.8)	
pN stage (n, %)				0.284
N0	59 (93.6)	44 (95.7)	15 (88.2)	
N1	4 (6.3)	2 (4.3)	2 (11.8)	
Surgical procedure				–
RN	36 (57.1)	22 (47.8)	14 (82.4)	
NSS	27 (42.9)	24 (52.2)	3 (17.6)	
Necrosis				**0.049**
Negative	48 (76.2)	38 (82.6)	10 (58.8)	
Positive	15 (23.8)	8 (17.4)	7 (41.2)	
Perinephric fat invasion (n, %)				0.926
Negative	59 (93.7)	43 (93.5)	16 (94.1)	
Positive	4 (6.3)	3 (6.5)	1 (5.9)	
Microvascular invasion (n, %)				0.540
Negative	62 (98.4)	45 (97.8)	17 (100)	
Positive	1 (1.6)	1 (2.2)	0 (0)	
Multiple eAML				0.800
Single	60 (95.2)	44 (95.7)	16 (94.1)	
Multiple	3 (4.8)	2 (4.3)	1 (5.9)	
Epithelioid cells (%, average ± SD)	85.6 ± 4.1	85.2 ± 4.2	86.8 ± 3.9	0.187
Nuclear atypia (n, %)				**0.005**
Mild	14 (22.2)	9 (19.6)	5 (29.4)	
Moderate	40 (63.5)	34 (73.9)	6 (35.3)	
Severe	9 (14.3)	3 (6.5)	6 (35.3)	
Mitotic count (n, average ± SD)	1.6 ± 1.9	0.7 ± 0.6 (range 0–2)	3.6 ± 2.2 (range 1–7)	** < 0.001**
Atypical mitoses (n, %)				** < 0.001**
Absence	47 (74.6)	41 (89.1)	6 (35.3)	
Presence	16 (25.4)	5 (11.9)	11 (64.7)	
Multinucleated giant cells (n, %)				0.071
Absence	34 (54.0)	28 (60.9)	6 (35.3)	
Presence	29 (46.0)	18 (39.1)	11 (64.7)	

RN, radical nephrectomy; NSS, Nephron-sparing surgery

*p value less than 0.05 was considered statistically significant and marked in bold

The average percentage of the epithelioid component was 85.6% (range 80–95%) (Fig. 1B). A total of 22.2% (14/63) cases revealed mild nuclear atypia, while 63.5% (40/63) exhibited moderate nuclear atypia and 14.3% (9/63) showed severe nuclear atypia. Importantly, elevated nuclear atypia was significantly correlated with malignancy (*p* = 0.005). Compared with cells displaying mild and moderate nuclear atypia, cells with severe nuclear atypia were characterized by their larger size and abundant eosinophilic cytoplasm, and nuclear polymorphism (Fig. 1C, D). Moreover, the mitotic count was 0–2 per 10 HPFs in the no recurrence/metastasis cohort, with an average mitotic count of 0.7, and 1–7 per 10 HPFs in the recurrence/metastasis cohort, with an average mitotic count of 3.6 (*p* < 0.001). In addition, 25.4% (16/63) cases displayed atypical mitotic figures (Fig. 1E), and the presence of atypical mitoses was significantly related to malignant behavior (*p* < 0.001). Multinucleated giant cells were observed in 46.0% (29/63) of cases (Fig. 1F), but the presence of multinucleated giant cells was not significantly correlated with malignancy (*p* = 0.071).

### Immunohistochemical features

**Fig. 2:**
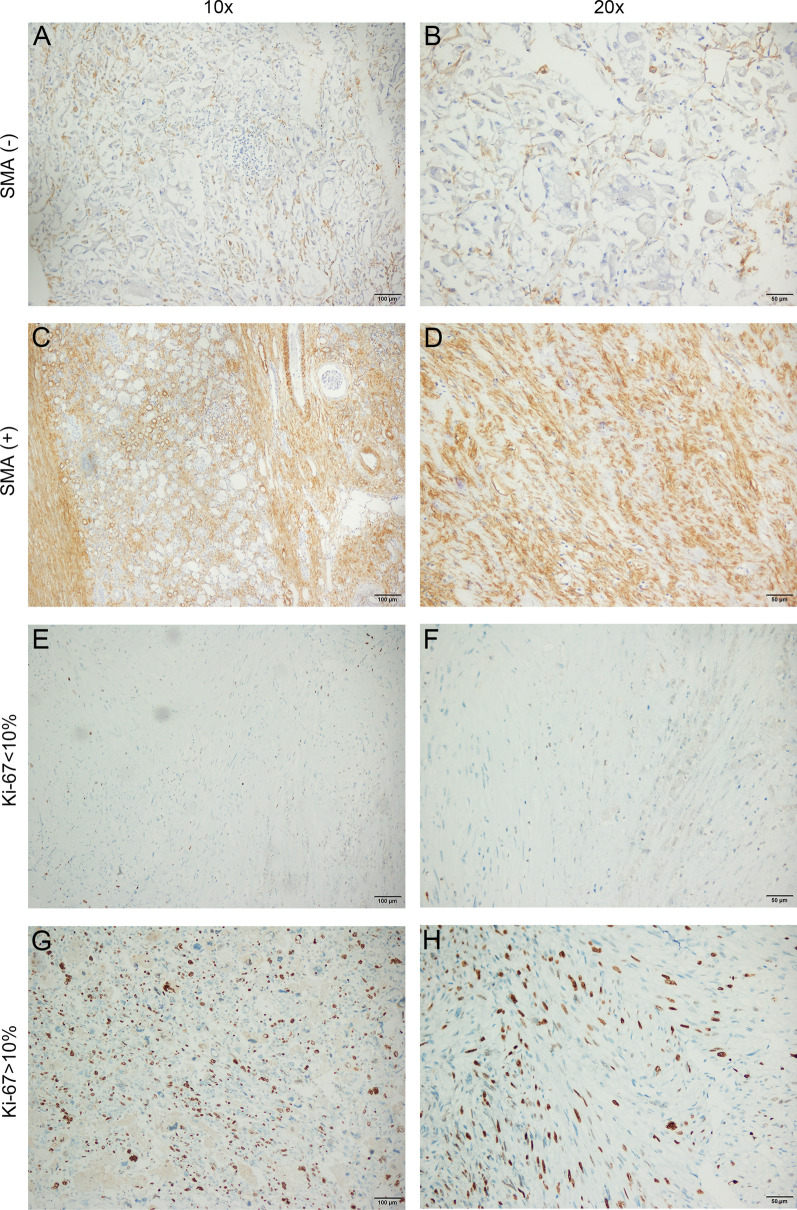
Immunohistochemical features of renal eAML. Immunoperoxidase staining. The overall immunohistochemistry (IHC) score of 0–3 was defined as negative staining and 4–12 as positive staining. **A**, **B** Metastatic renal eAML with negative SMA staining. **C**, **D** Non-metastatic renal eAML with diffuse positive SMA staining. **E**, **F** Non-metastatic renal eAML with less than 10% of cells showing nuclear staining with Ki-67. **G**, **H** Malignant renal eAML shows strong nuclear staining with Ki-67

The IHC staining was available for 57 renal eAML patients in the study cohort. All the cases exhibited positive staining of at least one melanocytic marker (HMB-45 or Melan-A). Negative SMA staining (IHC score 0–2) was significantly correlated with tumor recurrence/metastasis (*p* = 0.001), and the malignant cases displayed weak staining of SMA compared with cases with good outcomes. (Fig. 2A–D). In addition, patients with Ki-67 ≥ 10% were also significantly associated with recurrence/metastasis (*p* < 0.001), and malignant cases tended to show strong nuclear staining of Ki-67 relative to the cases with favorable prognoses (Fig. 2E–H). The chi-squared test revealed that other indexes were balanced in the distribution of categorical data, including HMB-45, Melan-A, PNL-2, desmin, CD34, AE1/AE3, CK7, vimentin, TFE3-OPT, PAX8, CD10, and S-100, as shown in Table 2.

**Table 2:** Comparison of immunohistochemical indexes of 57 renal eAML patients

Variable	Entire group (n = 57)	No recurrence/metastasis (n = 40)	Recurrence/metastasis (n = 17)	*P* value
HMB45 (−/ +)	7/50	4/36	3/14	0.421
Melan-A (−/ +)	11/46	7/33	4/13	0.598
SMA (-/ +)	14/43	5/35	9/8	**0.001**
PNL-2 (−/ +)	21/36	13/27	8/9	0.297
Desmin (−/ +)	43/14	31/9	12/5	0.579
CD34 (−/ +)	44/13	33/7	11/6	0.143
AE1/AE3 (−/ +)	53/4	36/4	17/0	0.176
CK7 (−/ +)	51/6	35/5	16/1	0.456
Vimentin (−/ +)	17/40	13/27	4/13	0.498
TFE3-OPT (−/ +)	42/15	30/10	12/5	0.729
PAX8 (−/ +)	49/8	35/5	14/3	0.609
CD10 (−/ +)	49/8	36/4	13/4	0.179
S-100 (−/ +)	30/27	19/19	11/8	0.574
Ki-67 (n, %)				** < 0.001**
< 10%	40 (70.2)	34 (89.5)	6 (31.6)	
≥ 10	17 (29.8)	4 (10.5)	13 (68.4)	

*p value less than 0.05 was considered statistically significant and marked in bold

### Prognostic factors for PFS and OS

The median PFS and OS of the total cohort were not reached during the follow-up period (Additional file 1: Fig. S1A, B). Patients with tumor size > 7 cm were significantly correlated with shorter PFS (*p* = 0.0061), but not significantly correlated with OS (*p* = 0.053) (Additional file 1: Fig. S1C, D). Besides, patients with pT3-pT4 also significantly correlated with poor PFS (*p* = 0.0044) and OS (*p* = 0.0022) (Additional file 1: Fig. S1E, F). In addition, presence of atypical mitoses (PFS: *p* < 0.0001, OS: *p* = 0.0085), presence of necrosis (PFS: *p* = 0.0428, OS: *p* = 0.0293), severe nuclear atypia (PFS: *p* < 0.0001, OS: *p* < 0.0001) and mitotic count >  = 2 (PFS: *p* < 0.0001, OS: *p* = 0.0174) were significantly correlated with both shorter PFS and OS (Additional file 1: Fig. S3G-N). Survival curves also demonstrated that patients with Ki-67 ≥ 10% were significantly correlated with poorer PFS (HR = 13.38, *p* < 0.0001) and OS (HR = 15.15, *p* = 0.0005) (Additional file 1: Fig. S1O, P). Besides, the survival analysis also indicated that patients with negative SMA staining (IHC score 0–2) had shorter PFS (HR = 4.59, *p* = 0.0002) and shorter OS (HR = 16.96, *p* < 0.0001) (Additional file 1: Fig. S1Q, R).

**Fig. 3:**
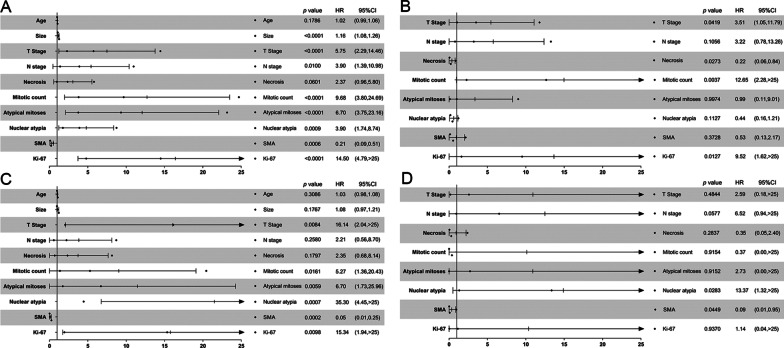
Univariate and multivariate Cox regression analysis. Univariate Cox regression analysis showed that tumor size (*p* < 0.0001), pT stage (*p* < 0.0001), pN stage (*p* = 0.0100), mitotic count (*p* < 0.0001), atypical mitoses (*p* < 0.0001), nuclear atypia (*p* = 0.0009), SMA expression (*p* = 0.0006) and percentage of Ki-67 (*p* < 0.0001) were significantly correlated with PFS (**A**), and pT stage (*p* = 0.0084), mitotic count (*p* = 0.0161), atypical mitoses (*p* = 0.0059), nuclear atypia (*p* = 0.0007), SMA expression (*p* = 0.0002) and percentage of Ki-67 (*p* = 0.0098) were significantly correlated with OS (**C**). In multivariate Cox regression analysis, pT stage (*p* = 0.0419), necrosis (*p* = 0.0273), mitotic count (*p* = 0.0037) and percentage of Ki-67 (*p* = 0.0127) were still significantly correlated with PFS (**B**), and nuclear atypia (*p* = 0.0283), SMA expression (*p* = 0.0449) were significantly correlated with OS (**D**)

Univariate and multivariate Cox regression analyses were performed on 57 eAML patients from the our insititution. The median follow-up was 49.5 months (range 8.9–102.5 months), and during the entire follow-up duration, 16 patients exhibited desease progression and seven patients died. As shown in Fig. 3A–D, in univariate Cox regression analysis, traditional prognostic factors, especially the pT stage were significantly correlated with PFS (*p* < 0.0001) and OS (*p* = 0.0084), but the pN stage was only significant for PFS (*p* = 0.0100). In addition, mitotic count (PFS: *p* < 0.0001; OS: *p* = 0.0161), atypical mitoses (PFS: *p* < 0.0001; OS: *p* = 0.0059), nuclear atypia (PFS: *p* = 0.0009; OS: *p* = 0.0007) were also significantly correlated with poor PFS and OS. Tumor size (*p* < 0.001) and necrosis were only significant for PFS. Importantly, SMA and Ki-67 were markedly associated with poor PFS (SMA: *p* = 0.0006; Ki-67: *p* < 0.0001) and OS (SMA: *p* = 0.0002; Ki-67: *p* = 0.0098). In the multivariate Cox regression analysis, pT stage (*p* = 0.042), necrosis (*p* = 0.027), mitotic count (*p* = 0.004) and Ki-67 (*p* = 0.013) were significantly correlated with poor PFS, and nuclear atypia (*p* = 0.028) and SMA (*p* = 0.045) were significantly correlated with OS.

### Construction of prognostic prediction model

**Fig. 4:**
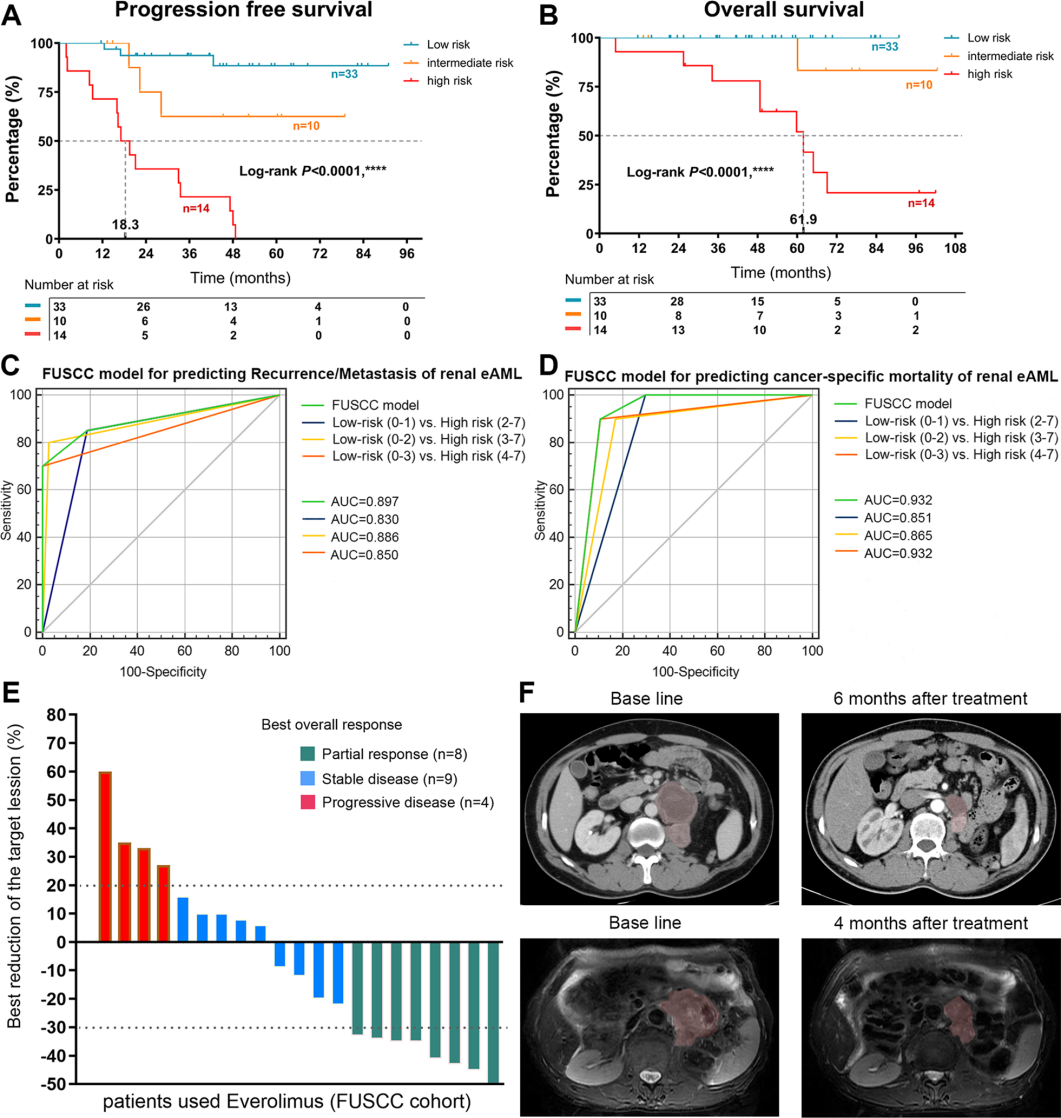
Construction of FUSCC cohort and drug efficacy. Patients in high risk group shows both poor PFS (**A**) and OS (**B**) than patients in low and intermediate risk group (*p* < 0.0001). **C**, **D** ROC curves indicates that the FUSCC model has high sensitivity and specificity to predict the recurrence/metastasis risk (AUC = 0.897) and cancer specific mortality (AUC = 0.932) of renal eAML patients. **E** The waterfall plot shows the best efficacy and reduction of the target lesion after Everolimus targeted treatment in 21 renal eAML patients with recurrence/metastasis, of which, the best response for Everolimus targeted treatment was 8/21 (38.1%) PR, 9/21 (42.9%) SD and 4/21 (19.0%) PD. **F** The representative imaging figures from one PR case (left: baseline CT imaging; right: 6 months after treatment) and one case of SD (right: baseline MRI imaging; right: 4 months after treatment

We selected significant indicators to establish a prognostic prediction model of renal eAML. After assessment of the prognostic markers of renal eAML, pT3-pT4, presence of necrosis, mitotic count ≥ 2; presence of atypical mitoses; severe nuclear atypia, SMA negative, Ki-67 ≥ 10% were considered to be risk factors for desease progression of renal eAML. Patients who had 0–1 risk factor were included in the low-risk group, patients with 2–3 of the risk factors were included in the intermediate-risk group, and patients with 4–7 risk factors were included in the high-risk group (Additional file 2: Table S1). Survival curves also indicated that the PFS and OS of the three prognostic groups differed significantly (*p* < 0.0001). The median PFS and OS of the low and intermediate-risk groups were not reached, which were much better than the high-risk group (median PFS:18.3; median OS: 61.9) (Fig. 4A, B). Overall, the stratification model sufficiently discriminated recurrence/metastasis (AUC = 0.897) and cancer-specific mortality (AUC = 0.932) of renal eAML patients in different risk groups. (Fig. 4C, D).

### Treatment

In the recurrence/metastasis group, four (6.0%) cases had confirmed distant metastasis through preoperative systemic imaging and confirmed by biopsy of the primary tumor at the time of diagnosis. Partial nephrectomy was performed in 27 (42.9%) cases and radical nephrectomy in 36 (57.1%) cases. Among patients undergoing protocol surgery, recurrence/metastasis occurred in 17 (27.0%) patients in the follow-up period with a mean time to recurrence/metastasis of 22.2 months. Imaging examination and puncture biopsy confirmed that the most frequent metastatic site was lung 12 (57.1%), as well as 8 (38.1%) cases with recurrence. All patients who occurred recurrence/metastasis during follow-up period (N = 17), as well as patients who ouccered metastasis at the time of initial diagnosis (N = 4) had received Everolimus (10 mg qd) targeted therapy. Based on radiology review, the best response for Everolimus treatment was 8/21 (38.1%) partial responses (PR), 9/21 (42.9%) stable disease (SD) and 4/21 (19.0%) progressive disease (PD) (Fig. 4E). Figure 4F is the representative imaging figures from one case of PR and one case of SD. Four PD cases were switched to second-line targeted therapy, of which two patients were treated with Axitinib, one patient was treated with Pazopanib, and one patient was treated with Sorafenib. One patient who received nephron sparing surgery had an operation area recurrence that was treated successfully with radical nephrectomy after partial response to Everolimus targeted therapy. One patient with lung metastasis received wedge resection of pulmonary metastasis after partial response. One patient received abdominal wall metastatic site resection after partial response to Everolimus targeted therapy. Treatment details were summarized in Table 3.

**Table 3:** Data of surgery and targeted therapy in recurrence/metastasis patients

Nos.	Age (years)/sex	Size	Surgical procedure	M at diagnosis	R/M site	First line therapy	Response	Time to R/M (mo)	Second line therapy	Survival status
1	31/F	11	Biopsy	Yes	Lung, liver, retroperitoneum	Everolimus	SD			Dead
2	44/F	20	RN	No	Lung	Everolimus	PD	32.8	Pazopanib	Alive
3	36/F	13	RN	No	Lung	Everolimus	SD	30.5		Alive
4	65/M	17	RN	No	Right paralumbar fossa	Everolimus	PR	20.7		Dead
5	29/M	13	RN	No	Lung, left paralumbar fossa	Everolimus	PR	7.4		Alive
6	62/F	5	RN	No	Lung, liver, lymph node, bone	Everolimus	SD	47.7		Dead
7	30/F	16	RN	No	Lung, lymph node	Everolimus	PD	16.6	Sorafenib	Dead
8	61/M	12.5	Biopsy	Yes	Lung, liver, bone	Everolimus	PD		Axitinib	Dead
9	44/F	9	RN	No	Lung, lymph node	Everolimus	PR	46.5	PWR	Alive
10	28/M	15	RN	No	Liver, lymph node	Everolimus	PD	9.3	Axitinib	Dead
11	46/F	20	RN	No	Lung	Everolimus	PR	22.3		Alive
12	76/M	11	RN	No	Retroperitoneum	Everolimus	SD	33.5		Dead
13	46/M	13	RN	No	Left paralumbar fossa	Everolimus	SD	3.5		Alive
14	24/F	7.2	RN	No	Lung, lymph node	Everolimus	SD	2.7		Dead
15	44/M	13.4	RN	No	Left paralumbar fossa	Everolimus	PR	19.5		Alive
16	26/M	10.1	Biopsy	Yes	Bone, lymph node	Everolimus	PR			Alive
17	28/F	6	NSS	No	Right paralumbar fossa	Everolimus	PR	19.3	RN	Alive
18	52/F	4.3	NSS	No	Lung	Everolimus	SD	17.0		Alive
19	21/M	5.1	NSS	No	Right paralumbar fossa	Everolimus	SD	21.3		Alive
20	54/M	7.5	RN	No	Abdominal wall	Everolimus	PR	28.8	Operation	Alive
21	36/F	12	Biopsy	Yes	Lung	Everolimus	SD			Alive

M, metastasis; R/M, recurrence/metastasis; F, female; M, male; RN, radical nephrectomy; NSS, nephron sparing surgery; PR, partial response; SD, stable disease; PD, progressive disease; PWR, pulmonary wedge resection

## Discussion

To clarify the potential malignancy of eAML and investigate prognostic predictors for this disease, we studied the pathological characteristics and clinical outcomes of 67 eAML patients. More importantly, we established a risk model for eAML patients and accurately predicted the recurrence/metastasis risk. Our model provides a novel approach for the diagnosis and early intervention of eAML, a potentially malignant disease.

Renal eAML was once considered a hamartoma [18]. However, in subsequent years, several studies reported the malignant behavior of renal eAML, including local recurrence, distant metastasis, and death from the disease, as described in small case reports [19–21]. Nevertheless, because of the rarity of renal eAML cases, the clinicopathological characteristics, diagnosis, and clinical outcomes should be further investigated for better treatment. To evaluate the potential malignancy of this rare disease, Brimo et al. studied a series of 40 renal eAML cases and demonstrated that the presence of at least 70% atypical epithelioid cells, a mitotic count of ≥ 2 per 10 HPFs, atypical mitotic figures, and necrosis were prognostic factors for renal eAML [16]. Nese et al. also performed a similar study of 41 renal eAML cases and indicated that necrosis, tuberous sclerosis complex and/or concurrent AML, carcinoma-like growth, extrarenal extension and/or the involvement of the renal vein, and tumor size > 7 cm were prognostic factors for renal eAML [17]. In previous studies, many predictors of poor clinical outcomes were investigated but not unified.

Studies have shown that eAML is a potentially malignant neoplasm, with approximately 30% of cases exhibiting distant metastasis to lymph nodes, liver, lungs, and spine [22]. Similarly, there were 17 (25.6%) patients with metastasis in our cohort. Notably, Antonio et al. first reported a case of primary eAML of the adrenal gland in patients without evidence of tuberous sclerosis [23]. Therefore, because of its similarity upon imaging, eAML is easily misdiagnosed as renal cell carcinoma or sarcoma [24]. Furthermore, abnormal blood vessels and mature adipocytes are not obvious in eAML, and thus it is difficult to diagnose eAML using radiographic devices [25]. Hence, the diagnosis of eAML should be mainly based on the different proportions of epithelioid components as well as positive staining of HMB-45 or Melan-A. In our eAML study cohort, all patients were diagnosed with the pathological characteristics described above, and 57 of these patients were examined to investigate the potential value of IHC indexes.

Ki-67 was first identified as an antigen by Gerdes et al. in Hodgkin lymphoma cell nuclei [26]. Previous studies based on cell cycle analyses have illustrated that among the identified cell cycle markers, only Ki-67 is downregulated in the quiescent G_0_ phase while being highly expressed in the G_1_, S, and G_2_ phases [27]. In recent years, similar investigations have been undertaken in renal eAML, but the results were inconclusive. Ooi et al. demonstrated that Ki-67 was strongly positive in two eAML cases but negative in four classic AMLs [28]. Moreover, Xu et al. evaluated the use of Ki-67 as a prognostic predictor in six eAML patients and found that patients with positive expression of Ki-67 had a poorer prognosis [29]. Conversely, no significant difference in Ki-67 was identified between classic AML and eAML cases [30]. However, all these studies were based on small sample sizes, and hence it is necessary to further verify the role of Ki-67 as a prognostic indicator in eAML patients.

The advantage of our study is that we systemically analyzed clinicopathological and IHC features and clinical outcomes in an unprecedented number of renal eAML cases. Through this, we demonstrated that renal eAML patients with malignancy exhibited larger tumor size, advanced T/N stage, and necrosis. Immunohistochemically, we also revealed that negative SMA expression and Ki-67 ≥ 10% were significantly correlated with malignancy. Based on these features, we attempted to investigate the prognostic factors of renal eAML patients and found that Ki-67 ≥ 10% and negative SMA expression were correlated with poor PFS and OS in eAML patients. Next, we established a risk model for renal eAML, which incorporated the most common, representative and accurate pathological parameters as risk factors.

This study has some limitations. First, due to the nature of the retrospective study and the missing data of some patients, we cannot but accept all the biases of our study. Second, our study did not examine the underlying mechanism of Ki-67 and SMA in the tumor metabolism of renal eAML. Third, our risk model should be verified in other renal eAML cases.

## Supplementary Information

**Additional file 1. Supplementary Figure 1.** Correlation between clinical parameters and prognosis. Survival curves indicated the PFS (A) and OS (B) of total cohort. Patients with tumour size>7cm were significantly correlated with PFS (C) (HR=4.47, *p*=0.0061), but not significant in OS (D) (HR=4.61, *p*=0.053). Patients with pT3-pT4 stage were correlated with both shorter PFS (E) (HR=3.74, *p*=0.0044) and OS (F) (HR=10.27, *p*=0.0022). Patients with presence of atypical mitosis (G, H), presence of necrosis (I, J), severe nuclear atypia (K, L), and mitotic count >=2 (M, N) were significantly correlated with poor PFS (presence of atypical mitoses: HR=5.41, *p*<0.0001; necrosis: HR=3.01, *p*=0.0428; severe nuclear atypia: *p*<0.0001; mitotic count>=2: HR=7.56, *p*<0.0001) and OS (presence of atypical mitoses: HR=5.30, *p*=0.0085; necrosis: HR=3.85, *p*=0.0293; nuclear atypia: *p*<0.0001; mitotic count: HR=4.40, *p*=0.0174). (O, P) Patients with Ki-67 ≥10% were significantly correlated with poor PFS (HR=13.38; *p*<0.0001 and OS (HR=15.15; *p*=0.0005). (Q, R) Patients with negative SMA staining (IHC score 0-2) also exhibited shorter PFS (HR=4.59; *p*=0.0002) and OS (HR=16.96; *p*<0.0001).

**Additional file 2. Supplementary Table 1.** Distribution of prognostic risk factors in 57 renal eAML patient. Risk factors included: pT3 and pT4, presence of necrosis, mitotic count≥2; the presence of atypical mitoses; severe nuclear atypia, SMA negative, Ki-67≥10%. Low-risk group: including 0-1 risk factor, Intermediate-risk group: including 2-3 risk factors, High-risk group: including 4-7 risk factors. *p value less than 0.05 was considered as statistically significant and marked in bold.

## Data Availability

The datasets used and/or analyzed during the current study are available from the corresponding author on reasonable request.
