# Supplementary material for: ♣Evaluation of clinicopathological profiles and development of a risk model in renal epithelioid angiomyolipoma patients: a large-scale retrospective cohort study
Source: BMC Urol. 2022 Sep 12;22:148. doi: 10.1186/s12894-022-01101-9 (PMC9469541; doi:10.1186/s12894-022-01101-9)
Supplement: Supplementary file 1 — Additional file 1. Supplementary Figure 1. Correlation between clinical parameters and prognosis. Survival curves indicated the PFS (A) and OS (B) of total cohort. Patients with tumour size>7cm were significantly correlated with PFS (C) (HR=4.47, p=0.0061), but not significant in OS (D) (HR=4.61, p=0.053). Patients with pT3-pT4 stage were correlated with both shorter PFS (E) (HR=3.74, p=0.0044) and OS (F) (HR=10.27, p=0.0022). Patients with presence of atypical mitosis (G, H), presence of necrosis (I, J), severe nuclear atypia (K, L), and mitotic count >=2 (M, N) were significantly correlated with poor PFS (presence of atypical mitoses: HR=5.41, p<0.0001; necrosis: HR=3.01, p=0.0428; severe nuclear atypia: p<0.0001; mitotic count>=2: HR=7.56, p<0.0001) and OS (presence of atypical mitoses: HR=5.30, p=0.0085; necrosis: HR=3.85, p=0.0293; nuclear atypia: p<0.0001; mitotic count: HR=4.40, p=0.0174). (O, P) Patients with Ki-67 ≥10% were significantly correlated with poor PFS (HR=13.38; p<0.0001 and OS (HR=15.15; p=0.0005). (Q, R) Patients with negative SMA staining (IHC score 0-2) also exhibited shorter PFS (HR=4.59; p=0.0002) and OS (HR=16.96; p<0.0001). [file 12894_2022_1101_MOESM1_ESM.docx]

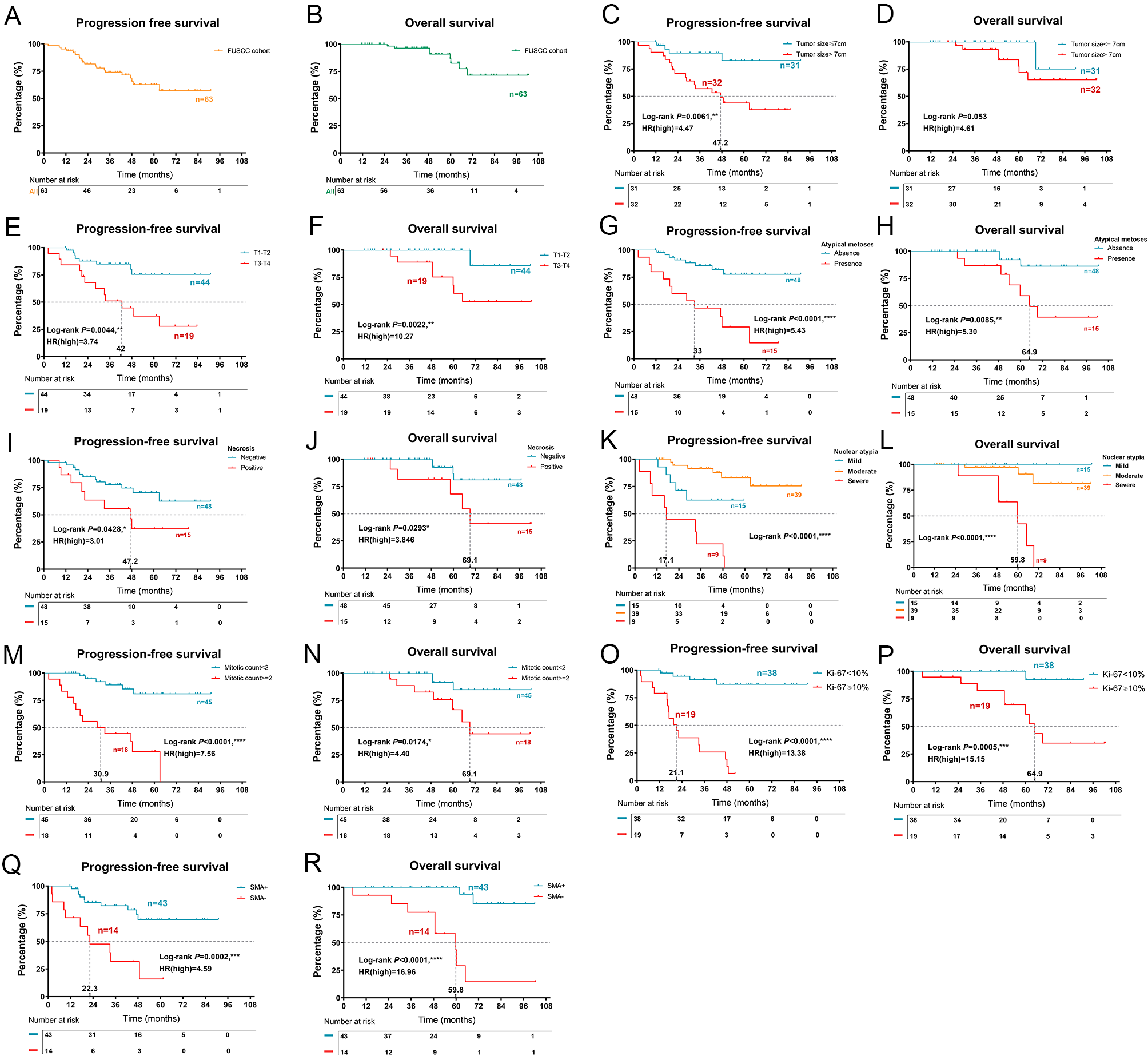


**Supplementary Figure 1. Correlation between clinical parameters and prognosis.** Survival curves indicated the PFS **(A)** and OS **(B)** of total cohort. Patients with tumour size>7cm were significantly correlated with PFS **(C)** (HR=4.47, *p*=0.0061), but not significant in OS **(D)** (HR=4.61, *p*=0.053). Patients with pT3-pT4 stage were correlated with both shorter PFS **(E)** (HR=3.74, *p*=0.0044) and OS **(F)** (HR=10.27, *p*=0.0022). Patients with presence of atypical mitosis **(G, H),** presence of necrosis **(I, J)**, severe nuclear atypia **(K, L)**, and mitotic count >=2 **(M, N)** were significantly correlated with poor PFS (presence of atypical mitoses: HR=5.41, *p*<0.0001; necrosis: HR=3.01, *p*=0.0428; severe nuclear atypia: *p*<0.0001; mitotic count>=2: HR=7.56, *p*<0.0001) and OS (presence of atypical mitoses: HR=5.30, *p*=0.0085; necrosis: HR=3.85, *p*=0.0293; nuclear atypia: *p*<0.0001; mitotic count: HR=4.40, *p*=0.0174). **(O, P)** Patients with Ki-67 ≥10% were significantly correlated with poor PFS (HR=13.38; p<0.0001) and OS (HR=15.15; p=0.0005). **(Q, R)** Patients with negative SMA staining (IHC score 0-2) also exhibited shorter PFS (HR=4.59; p=0.0002) and OS (HR=16.96; p<0.0001).
