# Supplementary material for: ♣Evaluation of clinicopathological profiles and development of a risk model in renal epithelioid angiomyolipoma patients: a large-scale retrospective cohort study
Source: BMC Urol. 2022 Sep 12;22:148. doi: 10.1186/s12894-022-01101-9 (PMC9469541; doi:10.1186/s12894-022-01101-9)
Supplement: Supplementary file 2 — Additional file 2. Supplementary Table 1. Distribution of prognostic risk factors in 57 renal eAML patient. Risk factors included: pT3 and pT4, presence of necrosis, mitotic count≥2; the presence of atypical mitoses; severe nuclear atypia, SMA negative, Ki-67≥10%. Low-risk group: including 0-1 risk factor, Intermediate-risk group: including 2-3 risk factors, High-risk group: including 4-7 risk factors. *p value less than 0.05 was considered as statistically significant and marked in bold. [file 12894_2022_1101_MOESM2_ESM.pdf]

Supplementary Table 1. Distribution of prognostic risk factors in 57 renal eAML patient

variable	Renal eAML patients No recurrence/metastasis (n=40)	Recurrence/metastasis (n=17)	χ^2	*P* value
Risk group			25.294	**<0.0001**
Low risk group (n, %)	31 (77.5)	2 (11.8)		
0 risk factor	22 (55.0)	0 (0)		
1 risk factor	9 (22.5)	2 (11.8)		
Intermediate risk group	6 (15.0)	4 (23.5)		
2 risk factors	5 (12.5)	2 (11.8)		
3 risk factors	1 (2.5)	2 (11.8)		
High-risk group (n, %)	3 (7.5)	11 (64.7)		
4 risk factors	2 (5.0)	3 (17.6)		
5 risk factors	0 (0)	2 (11.8)		
6 risk factors	0 (0)	3 (17.6)		
7 risk factors	1 (2.5)	3 (17.6)		

Risk factors included: pT3 and pT4, presence of necrosis, mitotic count≥2; the presence of atypical mitoses; severe nuclear atypia, SMA negative, Ki-67≥10%. Low-risk group: including 0-1 risk factor, Intermediate-risk group: including 2-3 risk factors, High-risk group: including 4-7 risk factors;

**p* value less than 0.05 was considered as statistically significant and marked in bold.
